# METTL3 Promotes Osteo/Odontogenic Differentiation of Stem Cells by Inhibiting miR-196b-5p Maturation

**DOI:** 10.1155/2023/8992284

**Published:** 2023-06-07

**Authors:** Xiao Han, Guoyue Li, Haoqing Yang, Chen Zhang, Yangyang Cao, Ning Wang, Lihua Ge, Zhipeng Fan

**Affiliations:** ^1^Beijing Key Laboratory of Tooth Regeneration and Function Reconstruction, Beijing Stomatological Hospital, School of Stomatology, Capital Medical University, Beijing 100050, China; ^2^Research Unit of Tooth Development and Regeneration, Chinese Academy of Medical Sciences, China

## Abstract

Mesenchymal stem cells (MSCs) have been considered a potential method for the regeneration of tooth and maxillofacial bone defects based on the multidirectional differentiation characteristics of MSCs. miRNAs have been found to play a key role in the differentiation of MSCs. However, its effectiveness still needs to be improved, and its internal mechanism is still unclear. In the present study, our data discovered that the knockdown of miR-196b-5p promoted alkaline phosphatase (ALP) activity assay, mineralization *in vitro*, and expressions of osteo/odontogenic differentiation markers DSPP and OCN and enhanced *in vivo* osteo/odontogenic differentiation of stem cells of the apical papilla (SCAPs). Mechanistically, the results indicated that METTL3-dependent N6-methyladenosine (m6A) methylation inhibited miR-196b-5p maturation by the microprocessor protein DGCR8. Moreover, miR-196b-5p indirectly negatively regulates METTL3 in SCAPs. Then, METTL3 was found to strengthen the ALP activity assay, mineralization, and expressions of osteo/dentinogenic differentiation markers. Taken together, our findings highlight the critical roles of the METTL3-miR-196b-5p signaling axis in an m6A-dependent manner in osteo/odontogenic differentiation of SCAPs, identifying some potential targets for tooth and maxillofacial bone defects.

## 1. Introduction

Tooth loss and maxillofacial bone defect caused by trauma, disease, or congenital dysplasia are common and frequently occurring oral diseases, which have serious impacts on the patients' chewing, speech, swallowing, and other functions. However, there are some strict indications and limitations in clinical repair methods, which cannot meet the needs of the patients. With the development of the technique, mesenchymal stem cells (MSCs) are widely used in the field of tissue regeneration and immunotherapy based on their excellent proliferation, multilineage differentiation, and immune regulation [1, 2]. Compared with nondental tissue-derived MSCs, dental tissue-derived MSCs have great advantages in the tissue regeneration of tooth and maxillofacial bone defects [3, 4]. As odontogenic MSCs, stem cells of the apical papilla (SCAPs) exhibited stronger osteo/odontogenic capacity [5, 6]. The capacity of SCAPs to differentiate allows them to function as the starting cells for MSC regenerative therapy.

MicroRNA (miRNA) is a class of noncoding single-stranded RNA molecule, which plays an important role in many aspects of biological functions [7–9]. Our previous study indicated that miR-196b-5p inhibited cell proliferation and affected the cell cycle of Wharton's jelly umbilical cord stem cells [10]. Mature miR-196b-5p is evolutionarily conserved in many species [11]. miRNA array data display that miR-196 is upregulated in chondrocytes suggesting that it may participate in human-induced pluripotent stem cell (hiPSC) cartilage differentiation [12]. Recently, Shi et al. found that miR-196b-5p regulated the mechanistic target of rapamycin complex 1 (mTORC1) and transforming growth factor-*β* (TGF-*β*) signaling by targeting tuberous sclerosis complex 1 (TSC1) and transforming growth factor-beta receptor 1 (TGF*β*R1) to promote adipogenic differentiation and adipogenesis of progenitor cells [13]. However, the role of miR-196b-5p in SCAPs was unclear.

m6A modification is an important posttranscriptional mRNA modification in mammals, which regulates a variety of biological processes [14, 15]. m6A methylation is dynamic and reversible, mainly including methyltransferase, demethyltransferase, and methylated recognition protein [16, 17]. In our previous data, we discovered that METTL3 was differentially expressed in the miR-196b-5p knockdown group and Consh group by SWATH-MS array [10]. Recently, studies revealed that m6A modification regulated the biosynthesis of miRNAs by participating in pri-miRNA processing or pre-miRNA splicing [18, 19]. Methyltransferase-like 3 (METTL3) was first discovered as a methyltransferase having a catalytic subunit, which was the main catalytic core [20]. Many studies have shown that the absence of METTL3 can cause abnormal m6A level and disrupt the normal lineage and differentiation of functional stem cells, leading to delayed neurogenesis, immune deficiency, and infertility [21, 22]. Han et al. reported that METTL3 accelerated the processing of pri-miR-221/222 by binding with DiGeorge syndrome critical region 8 (DGCR8) in bladder cancer cells [23]. In addition, Tian et al. discovered that the loss of METTL3 suppressed the osteogenic differentiation potential of bone mesenchymal stem cells (BMSCs) in 2019 [24]. However, the function of METTL3 in SCAP osteo/odontogenic differentiation is unknown.

In the present study, we investigate the role of miR-196b-5p on osteo/odontogenic differentiation of SCAPs. We revealed that the knockdown of miR-196b-5p promoted osteo/odontogenic differentiation of SCAPs *in vitro* and *in vivo*, and METTL3 enhanced osteo/odontogenic differentiation of SCAPs *in vitro*. Furthermore, mechanism studies indicated that METTL3 suppressed the maturation of miR-196b-5p by combining with DGCR8. In conclusion, METTL3 promotes osteo/odontogenic differentiation of SCAPs by suppressing miR-196b-5p maturation in an m6A-dependent manner.

## 2. Materials and Methods

### 2.1. Cell Culture

Dental tissue acquisition followed the guidelines approved by Beijing Stomatological Hospital of Capital Medical University (ethics approval no. 2011-02), and the patients had informed consent. SCAPs were isolated and cultured as shown in our previous study [25, 26]. In this study, 10 *μ*M cycloleucine (Cat No. 52528, MCE, China) was used to treat SCAPs.

### 2.2. Synthesis of miRNA and Construction

miR-196b-5p mimic, miR-196b-5p inhibitor, METTL3 short hairpin RNA (shRNA), LV3 shRNA (Consh), and METTL3 overexpressed construct were obtained from GenePharma (Suzhou, China). And LV3 shRNA (Consh) was a negative control for miR-196b-5p inhibitor, miR-196b-5p mimic, and METTL3 shRNA. For gene synthesis, human full-length METTL3 gene sequence was subcloned into the pQCXIN retroviral vector at the BamH1 and PacI restriction sites. Vector was negative control for METTL3 overexpression. Virus transfection was conducted as shown in our previous study [27] (LV3 shRNA (Consh), 5′CAGUACUUUUGUGUAGUACAA-3′; miR-196b-5p mimic, 5′-TAGGTAGTTTCCTGTTGTTGGG-3′; miR-196b-5p inhibitor, 5′- CCCAACAACAGGAAACTACCTA-3′; and METTL3 shRNA (METTL3sh), 5′- GCTGCACTTCAGACGAATTAT-3′).

### 2.3. Alkaline Phosphatase (ALP) and Alizarin Red Detection

ALP activity was examined with an ALP activity kit (Sigma-Aldrich, St. Louis, MO, USA). ALP activity, alizarin red staining (ARS), and quantitative calcium analysis (QCA) were conducted as described previously [25, 27].

### 2.4. RNA Isolation and Real-Time Quantitative RT-PCR (qRT-PCR)

Total RNA was isolated from SCAPs by TRIzol (Invitrogen). The levels of pri-miR-196b, miR-196b-5p, or mRNA in SCAPs were detected by using TaqMan™ pri-miRNA assay (Cat. No. 4427012, Thermo Fisher Scientific, USA) and TaqMan microRNA assay (Cat. No. 4427975, Thermo Fisher Scientific, USA). U6 or glyceraldehyde-3-phosphate dehydrogenase (GAPDH) was used to normalize pri-miRNA, miRNA, or mRNA levels. The primer sequences are listed in Table 1.

### 2.5. Western Blot

Total proteins were resolved from SCAPs, and SDS polyacrylamide gel tests were performed as described previously [25]. The primary antibodies in this study were METTL3 (Cat. No. 15073-1-AP, Proteintech, China), DGCR8 (Cat. No. 10996-1-AP, Proteintech, China), DSPP (Cat. No. bs10316R, Bioss, China), OCN (Cat. No. bs-4917R, Bioss, China), and GAPDH (Cat. No. G8795, Sigma-Aldrich).

### 2.6. Flow Cytometry Analysis of SCAPs

SCAPs at passage 3 were digested by 0.25% trypsin, washed twice by PBS, and then used as single cell suspension. Approximately 2.0 × 10^5^ cells were incubated with fluorescence-coupled antibodies for 30 min at 4°C and analyzed by flow cytometry (Calibur; BD Biosciences) with FlowJo 10. The antibodies involved are as follows: FITC anti-rat CD90 antibody (Cat. No. 206105, BioLegend, USA), PE anti-mouse CD105 (Cat. No. 120407, BioLegend, USA), APC anti-mouse CD146 (Cat. No. 134711, BioLegend, USA), FITC anti-mouse CD45 (Cat. No. 157607, BioLegend, USA), and FITC anti-mouse CD34 antibody (Cat. No. 343503, BioLegend, USA).

### 2.7. Coimmunoprecipitation (Co-IP) Assay

SCAPs were lysed with the IP lysis buffer (Invitrogen). Co-IP was performed as described previously [27]. The primary antibodies in this study were METTL3 (Cat. No. 15073-1-AP, Proteintech, China), DGCR8 (Cat. No. 10996-1-AP, Proteintech, China), and GAPDH (Cat. No. G8795, Sigma-Aldrich).

### 2.8. m6A RNA Immunoprecipitation Assay (MeRIP)

The m6A RNA binding assays, BersinBio™ Methylated RNA Immunoprecipitation Kit (Cat. No. Bes5203, China), were used. Briefly, fragmented RNA was incubated with magnetic beads bound to m6A antibody (Cat. No. Bes5203, China) for immunoprecipitation. Analyze the enrichment of m6A-mRNA by qRT-PCR and normalize the input.

### 2.9. RNA Immunoprecipitation Assay

The RIP assay was conducted by BersinBio™ RNA Immunoprecipitation Kit (Cat. No. Bes5101, China). Briefly, cell lysates were incubated with magnetic beads coupled with anti-DGCR8 (Cat. No. 10996-1-AP, Proteintech, China) or IgG (sc-2027, Santa Cruz Biotechnology). Finally, the RNAs were extracted for qRT-PCR and normalized to input.

### 2.10. Nude Mouse Transplantation and Immunohistochemical Staining

A total of 2.0 × 10^6^ SCAPs were mixed with 20 mg HA/tricalcium phosphate (Engineering Research Center for Biomaterials, Sichuan University, Chengdu, China) at 37°C for 2 h, and then, the mixture was subcutaneously transplanted into the back of nude mice (10-week-old female, nu/nu). After 8 weeks, the subcutaneous transplanted tissues were collected, fixed with 10% formalin, and decalcified in 10% EDTA. The tissues were stained with hematoxylin-eosin (HE). Immunohistochemical staining was performed as described previously [25]. The primary antibodies used were as follows: OCN antibody (Cat. No. bs-4917R, Bioss, China) and DSPP antibody (Cat. No. bs10316R, Bioss, China). Our research was carried out in accordance with the animal experiment regulations (Ethics Committee Agreement, Beijing Stomatological Hospital, ethics approval no. KQYY-201909-001).

### 2.11. Statistical Analysis

Statistical calculations were implemented using SPSS 10.0 statistical software (SPSS Inc., Chicago, IL, USA). Statistical significance was analyzed by Student's *t*-test or one-way ANOVA; *p* ≤ 0.05 was regarded as statistical significance.

## 3. Results

### 3.1. miR-196b-5p Suppressed the Osteo/Odontogenic Differentiation of SCAPs *In Vitro* and *In Vivo*

**Figure 1:**
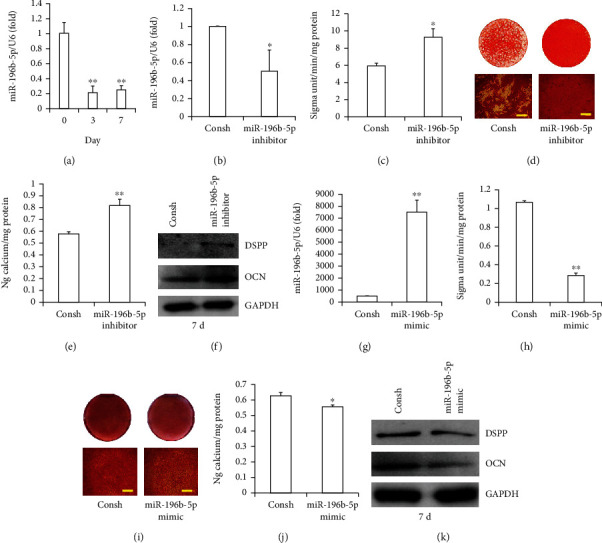
miR-196b-5p suppressed the osteo/odontogenic differentiation potential of SCAPs *in vitro*. (a) The mRNA expression of miR-196b-5p was detected by real-time RT-PCR after mineralization induction at 3 and 7 days. (b) Real-time RT-PCR showed the knockdown efficiency of miR-196b-5p in SCAPs. (c) ALP activity assay demonstrating that miR-196b-5p knockdown enhanced ALP activity in SCAPs. (d) Alizarin red staining and (e) quantitative calcium analysis results demonstrating that miR-196b-5p knockdown enhanced mineralization in SCAPs (scale bar: 50 *μ*m). (f) Western blot result demonstrating that the knockdown of miR-196b-5p upregulated the expression of DSPP and OCN in SCAPs. (g) Real-time RT-PCR showed the efficiency of miR-196b-5p overexpression in SCAPs. (h) ALP activity assay demonstrating that miR-196b-5p overexpression inhibited ALP activity in SCAPs. (i) Alizarin red staining and (j) quantitative calcium analysis results demonstrating that miR-196b-5p overexpression weakened mineralization in SCAPs (scale bar: 50 *μ*m). (k) Western blot result demonstrating that the overexpression of miR-196b-5p downregulated the expression of DSPP and OCN in SCAPs. U6 or GAPDH was used for standardization. Student's *t*-test or one-way ANOVA was used to analyze the statistical significance. All error bars signify standard deviations (*n* = 3). ^∗^*p* ≤ 0.05 and ^∗∗^*p* ≤ 0.01.

We examined MSC markers in SCAPs by flow cytometric analysis. The results showed that surface markers CD90, CD105, and CD146 were positively expressed, while CD34 and CD45 were negatively expressed in SCAPs (Supplementary Figure 1). To verify the role of miR-196b-5p on SCAP osteo/odontogenic differentiation, first, we discovered that the mRNA level of miR-196b-5p was downregulated at 3 and 7 days after osteo/odontogenic differentiation in SCAPs (Figure 1(a)). Then, we transduced miR-196b-5p inhibitor and Consh into SCAPs via lentiviral infection. After 3 days of 1 *μ*g/ml puromycin treatment, the efficiency of miR-196-5p knockdown was confirmed by qRT-PCR (Figure 1(b)). After 5 days of mineralization induction, ALP activity indicated that the knockdown of miR-196b-5p was heightened compared with the Consh group (Figure 1(c)). Then, ARS and QCA indicated that miR-196b-5p inhibitor enhanced mineralization compared with Consh (Figures 1(d) and 1(e)). Western blot (WB) assay showed that the expression of DSPP and OCN in miR-196b-5p inhibitor was strengthened compared with the control group at 7 days after osteo/odontogenic induction (Figure 1(f)). To further identify the function of miR-196b-5p on osteo/odontogenic differentiation of SCAPs, miR-196b-5p mimic with a lentiviral construct was transduced into SCAPs; qRT-PCR was used to confirm the overexpression efficiency (Figure 1(g)). ALP activity was suppressed after overexpression of miR-196b-5p in SCAPs (Figure 1(h)). Furthermore, ARS and QCA indicated that the overexpression of miR-196b-5p repressed mineralization *in vitro* in SCAPs compared with the Consh group (Figures 1(i) and 1(j)). After 7 days of mineralization induction, the WB assay indicated that miR-196b-5p overexpression decreased the expression of DSPP and OCN (Figure 1(k)).

**Figure 2:**
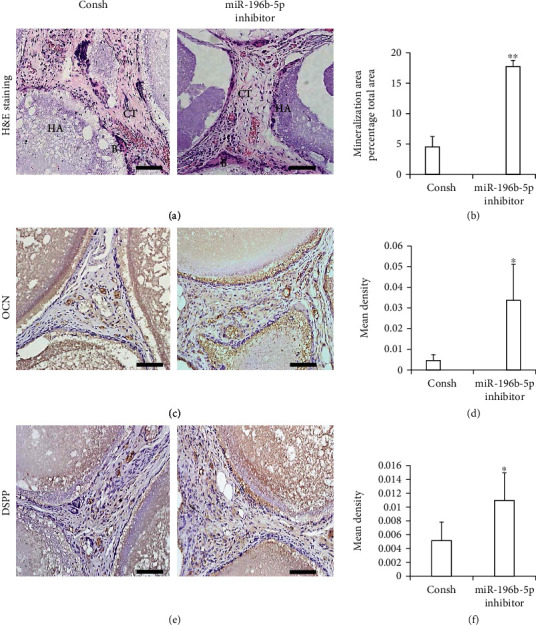
Knockdown of miR-196b-5p promoted the osteo/odontogenic of SCAPs *in vivo*. (a) HE staining results showed bone-/dentin-like tissue formation. Scale bar: 100 *μ*m. B: bone-/dentin-like tissues; HA: hydroxyapatite tricalcium carrier; CT: connective tissue. (b) Quantitative measurement of HE staining results. Immunohistochemical staining and quantitative analysis of DSPP (c, d) and OCN (e, f). Scale bar: 100 *μ*m. Student's *t*-test was performed to determine statistical significance. All error bars represent the standard deviation (*n* = 6). ^∗^*p* ≤ 0.05 and ^∗∗^*p* ≤ 0.01.

Then, SCAP-Consh and SCAP-miR-196b-5p inhibitor cells were transplanted subcutaneously into nude mice. We discovered that miR-196b-5p inhibitor formed more bone-/dentin-like tissues (Figures 2(a) and 2(b)). Moreover, immunohistochemical staining and quantitative measurement results displayed that the expressions of DSPP and OCN were higher in the SCAP-miR-196b-5p inhibitor group than those in the Consh group (Figures 2(c)–2(f)).

### 3.2. METTL3 Enhanced the Osteo/Odontogenic Differentiation of SCAPs

**Figure 3:**
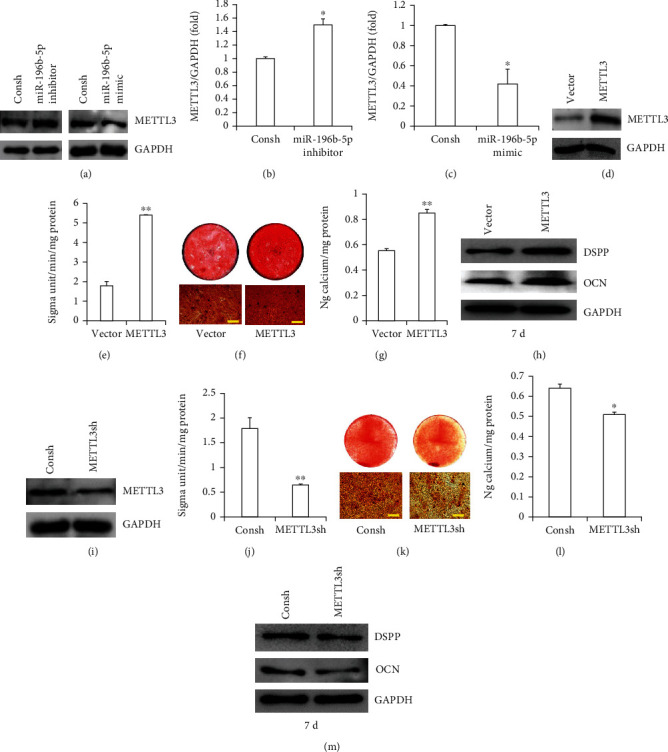
METTL3 promoted the osteo/odontogenic differentiation of SCAPs. (a–c) The protein and mRNA expressions of METTL3 were confirmed by western blot and real-time RT-PCR in miR-196b-5p mimic and in miR-196b-5p inhibitor. (d) The overexpression efficiency of METTL3 was confirmed by western blot. (e) ALP activity assay indicated that METTL3 overexpression enhanced ALP activity in SCAPs. (f) Alizarin red staining and (g) quantitative calcium analysis results demonstrated that METTL3 overexpression enhanced mineralization in SCAPs (scale bar: 50 *μ*m). (h) Western blot result demonstrating that METTL3 overexpression upregulated the expression of DSPP and OCN in SCAPs. (i) The knockdown efficiency of METTL3 was confirmed by western blot. (j) ALP activity assay indicated that METTL3 knockdown inhibited ALP activity in SCAPs. (k) Alizarin red staining and (l) quantitative calcium analysis results demonstrating that METTL3 knockdown inhibited mineralization in SCAPs (scale bar: 50 *μ*m). (m) Western blot result demonstrating that METTL3 knockdown downregulated the expression of DSPP and OCN in SCAPs. GAPDH was used for standardization. Student's *t*-test was used to analyze the statistical significance. All error bars signify standard deviations (*n* = 3). ^∗^*p* ≤ 0.05 and ^∗∗^*p* ≤ 0.01.

Subsequently, western blot and the real-time RT-PCR assay indicated that the expression of METTL3 was reduced in miR-196b-5p mimic but added in miR-196b-5p inhibitor (Figures 3(a)–3(c)). To explore the role of METTL3 on SCAP osteo/odontogenic differentiation, we inserted METTL3 sequences into lentiviral vectors and transferred them to SCAPs by lentiviral infection. The efficiency of METTL3 overexpression was detected by western blot (Figure 3(d)). ALP activity was heightened after METTL3 overexpression in SCAPs (Figure 3(e)). ARS and QCA showed that overexpression of METTL3 increased the mineralization of SCAPs (Figures 3(f) and 3(g)). After 7 days of mineralization induction, the WB assay suggested that the expression of DSPP and OCN in METTL3 overexpression was enhanced compared with that in the Vector group (Figure 3(h)). Then, we transduced METTL3 shRNA into SCAPs to inhibit METTL3 expression with lentivirus infection. WB was used to confirm the efficiency (Figure 3(i)). METTL3 knockdown depressed the ALP activity (Figure 3(j)). ARS and QCA results showed that the knockdown of METTL3 lowered the mineralization of SCAPs (Figures 3(k) and 3(l)). Furthermore, the WB assay showed that the expression of DSPP and OCN in METTL3 knockdown was decreased compared with that in the Consh group (Figure 3(m)).

### 3.3. METTL3 Interacted with DGCR8 and Modulated the miR-196b-5p Maturation in an m6A-Dependent Manner

**Figure 4:**
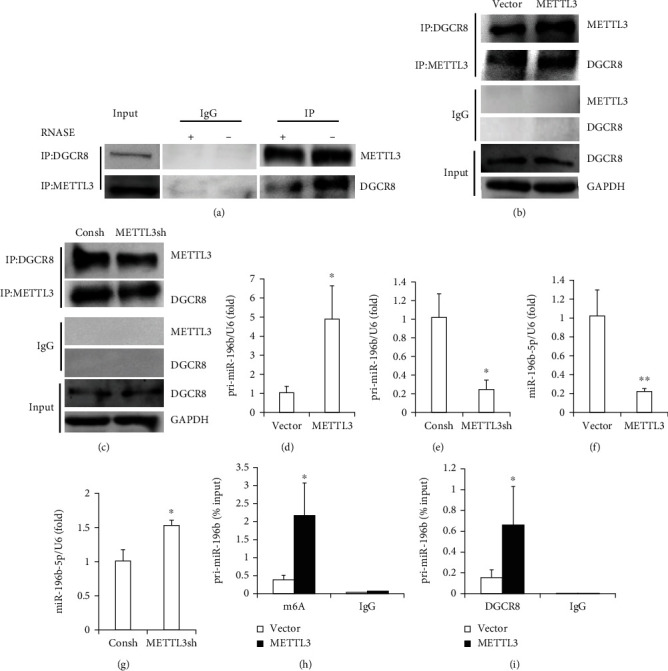
METTL3-dependent m6A methylation regulates miR-196b-5p maturation by the microprocessor protein DGCR8. (a) Co-IP assay showed that the METTL3 interacted with DGCR8 by western blot in SCAPs. (b, c) Co-IP of DGCR8, METTL3, and associated RNAs from METTL3 overexpression cells and METTL3 knockdown cells. Western blot using the DGCR8 and METTL3 antibodies and IgG was used as the control for the IP. (d, e) The expression of pri-miR-196b was verified by qRT-PCR in METTL3 overexpression and knockdown cells. (f, g) The expression of miR-196b-5p was verified by qRT-PCR in METTL3 overexpression and knockdown cells, respectively. (h) The detection of pri-miR-196b m6A modification level by MeRIP in control or METTL3 overexpression cells followed by qRT-PCR. (i) Detection of pri-miR-196b binding to DGCR8 by immunoprecipitation of DGCR8-associated RNA from control and METTL3 overexpression cells followed by qRT-PCR. U6 was used as an internal control for pri-miR-196b and miR-196b-5p. Student's *t*-test was used to analyze the statistical significance. All error bars signify standard deviations (*n* = 3). ^∗^*p* ≤ 0.05 and ^∗∗^*p* ≤ 0.01.

Subsequently, we made an attempt to predict whether there were binding sites between miR-196b-5p and the 3′UTR of METTL3 through the three kinds of software (TargetScan, miRDB, and miRbase). Unfortunately, no binding site was found. These findings showed that miR-196b-5p indirectly and negatively regulated the expression of METTL3. Then, to evaluate whether METTL3 was required for DGCR8 to participate in pri-miRNA processing in SCAPs, we first conducted coimmunoprecipitation assays. The results indicated that METTL3 interacted with DGCR8. And RNase attenuated this coprecipitation, showing that the coprecipitation between METTL3 and DGCR8 may be partially mediated by RNAs (Figure 4(a)). Furthermore, Co-IP assay showed that pri-miRNAs bound with DGCR8 increased in overexpression of METTL3 cells (Figure 4(b)), while pri-miRNAs bound by DGCR8 decreased in knockdown of METTL3 cells (Figure 4(c)). The results confirmed that METTL3 regulated pri-miRNA processing by binding to DGCR8 for pri-miRNA recognition and binding. Then, we detected the expression of pri-miR-196b and miR-196b-5p in the overexpression of METTL3 or knockdown of METTL3 cells. Our data demonstrated that expression of pri-miR-196b was increased in overexpression of METTL3 and decreased in the knockdown of the METTL3 group (Figures 4(d) and 4(e)). However, miR-196b-5p was decreased in the overexpression of METTL3 and increased in the knockdown of the METTL3 group (Figures 4(f) and 4(g)). Moreover, MeRIP revealed that the amount of pri-miR-196b modified by m6A was significantly increased in METTL3 overexpression (Figure 4(h)). Furthermore, we also revealed that the number of pri-miR-196b bound by DGCR8 was upregulated in METTL3 overexpression by an RIP assay (Figure 4(i)). Taken together, all these results indicated that METTL3 in an m6A methylation regulated miR-196b-5p processing by DGCR8.

Subsequently, SCAPs were transfected with the lentivirus and retrovirus of Consh+Vector, Consh+METTL3, and METTL3+miR-196b-5p mimic, and it was further verified that METTL3 affected the osteo/odontogenic differentiation of SCAPs by miR-196b-5p. Then, the ALP activity in the SCAP-Consh+METTL3 group was higher than that in the SCAP-Consh+Vector and METTL3+miR-196b-5p mimic group. ARS results revealed that the mineralization in the SCAP-Consh+METTL3 group was higher than that in the SCAP-Consh+Vector and METTL3+miR-196b-5p mimic group. The results indicated that miR-196b-5p mimic could attenuate osteogenic differentiation induced by METTL3 overexpression (Supplementary Figure 2).

### 3.4. Cycloleucine Increased miR-196b-5p Levels and Suppressed the Osteo/Odontogenic Differentiation of SCAPs

**Figure 5:**
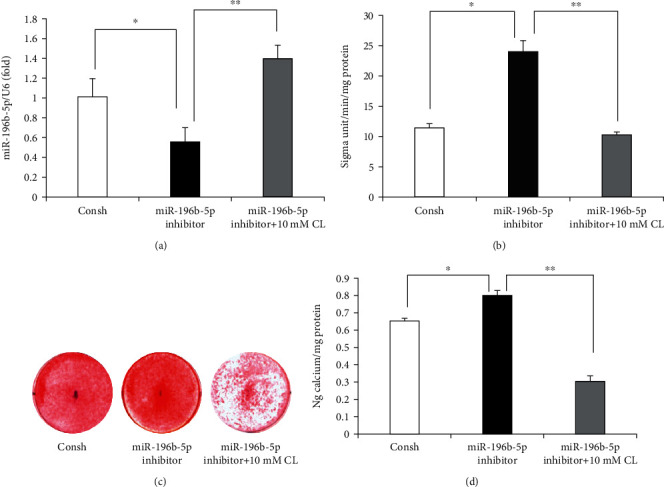
Cycloleucine upregulated miR-196b-5p levels and inhibited the osteo/odontogenic differentiation of SCAPs. (a) Effect of cycloleucine (10 mM) on the expression of miR-196b-5p. (b) ALP activity assay indicated that 10 mM CL repressed the knockdown of miR-196b-5p promoting ALP activity of SCAPs. (c) Alizarin red staining and (d) quantitative calcium analysis results demonstrating that 10 mM CL suppressed the enhanced mineralization by knockdown miR-196b-5p. One-way ANOVA was used to analyze the statistical significance. All error bars signify standard deviations (*n* = 3). ^∗^*p* ≤ 0.05 and ^∗∗^*p* ≤ 0.01.

Cycloleucine (CL) was applied to detect the relationship between m6A modification and miR-196b-5p in osteo/odontogenic differentiation. CL is a competitive and reversible methionine adenosyltransferase inhibitor, which inhibits methylation by reducing the concentration of S-adenosylmethionine. Previous studies showed that 20 and 40 mM CL significantly inhibited cell growth, while 10 mM concentration had no effect [28–30]. Therefore, 10 mM CL was used in this experiment. The study showed that 10 mM CL significantly increased the expression of miR-196b-5p compared with a knockdown miR-196b-5p group (Figure 5(a)). Then, we found that 10 mM CL repressed the knockdown of miR-196b-5p promoting ALP activity of SCAPs (Figure 5(b)). Furthermore, ARS and QCA showed that 10 mM CL suppressed the enhanced mineralization by the underexpression of miR-196b-5p (Figures 5(c) and 5(d)).

## 4. Discussion

MSCs are widely used in the regeneration of tooth and maxillofacial bone defects based on the multidirectional differentiation properties of MSCs. Increasingly, miRNAs have been proven to modulate the osteogenic differentiation of SCAPs [31–33]. In our studies, the functional studies revealed that miR-196b-5p inhibitor enhanced ALP activity, mineralization, and the expression of osteo/odontogenic markers including DSPP and OCN *in vitro* and increased bone-like tissue formation *in vivo*. According to the above research results, we deduced that miR-196b-5p acts as an osteogenic/odontogenic inhibitor in SCAPs.

Some mechanisms by which miR-196b-5p regulates gene expression have been known [34–36]. In our previous data, we discovered that METTL3 was differentially expressed in the miR-196b-5p knockdown group and Consh group by SWATH-MS array [10]. Then, the array displayed that miR-196b-5p negatively modulated METTL3 in mRNA and protein levels. Subsequently, we made an attempt to predict whether there were binding sites between miR-196b-5p and the 3′UTR of METTL3 through the three kinds of software (TargetScan, miRDB, and miRbase). Unfortunately, no binding site was found. These findings showed that miR-196b-5p indirectly and negatively regulated the expression of METTL3. METTL3 is known to be the first m6A methyltransferase to be discovered [20]. Recently, studies have uncovered a close and complex relationship of METTL3-mediated m6A modification with stem cell regulation [37, 38]. In the current study, we discovered that METTL3 promoted osteo/odontogenic differentiation of SCAPs. Our data is according to the findings of Tian et al. [24].

m6A modification is involved in regulating many aspects of biological functions, such as cell behavior, development, and disease [39, 40]. m6A modification has been found to play a part in regulating miRNA biosynthesis. As Alarcón et al. first reported in 2015, methylase METTL3 regulated miRNA biosynthesis [18]. Until 2019, Han et al. demonstrated this mechanism in bladder cancer cells, suggesting that METTL3 accelerated the maturation of miR-221/222 by interacting with DGCR8 [23]. Recently, Yan et al. have revealed that METTL3 inhibited the expression of miR-320 in BMSCs [41]. In this study, our results showed that METTL3 coprecipitated with DGCR8. When RNase was added, the coprecipitation of METTL3 and DGCR8 was weakened, indicating that the bound between METTL3 and DGCR8 may be partly mediated by RNA in SCAPs. Subsequently, we found a significantly increased DGCR8-bound RNA in METTL3 overexpression and a significantly reduced knockdown of METTL3. Then, we observed that the overexpression of METTL3 added the expression of pri-miR-196b while reducing the expression of miR-196b-5p, while knockout of METTL3 gave the opposite result. Moreover, RIP and MeRIP assays showed that METTL3 could regulate pri-miR-196b process in an m6A-dependent manner through DGCR8. Our findings are consistent with the previous studies [18, 41]. Furthermore, METTL3 overexpression was cotransfected with miR-196b-5p mimic to induce differentiation *in vitro*. The results showed that miR-196b-5p mimic attenuated osteo/odontogenic differentiation promoted by the METTL3 overexpression in SCAPs.

Cycloleucine (CL) was applied to detect the relationship between m6A modification and miR-196b-5p in osteo/odontogenic differentiation. CL is a competitive and reversible methionine adenosyltransferase inhibitor, which inhibits methylation by reducing the concentration of S-adenosylmethionine. In previous studies, CL was shown to be toxic to cells at high concentrations and reduced m6A levels in a dose-dependent manner. Previous studies showed that 20 and 40 mM CL significantly inhibited cell growth, while 10 mM concentration had no effect [28–30]. Therefore, 10 mM CL was used in this experiment. The current study indicated that 10 mM CL upregulated the mRNA levels of miR-196b-5p. Furthermore, 10 mM CL could block the miR-196b-5p knockdown-induced osteo/odontogenic differentiation in SCAPs. The findings suggested that METTL3 promoted osteo/odontogenic differentiation of SCAPs in an m6A-dependent manner.

## 5. Conclusion

In conclusion, our data indicated that miR-196b-5p inhibited the osteo/odontogenic differentiation of SCAPs, and METTL3 promoted the osteo/odontogenic differentiation of SCAPs in an m6A-dependent manner. And METTL3 suppressed miR-196b-5p maturation in SCAPs. Our findings highlight the key role of the METTL3-miR-196b-5p signaling axis in an m6A-dependent manner in osteo/odontogenic differentiation of SCAPs, which may be a potential target gene to promote MSC osteo/odontogenic differentiation and tissue regeneration.

## Figures and Tables

**Table 1:** Sequences used in the study.

Gene symbol	Sequence (5′-3′)
miR-196b-5p	F: GCGTAGGTAGTTTCCTG
miR-196b-5p	R: GAGCAGGCTGGAGAA
U6	F: GCTTCGGCAGCACATATACT
U6	R: GAGCAGGCTGGAGAA
METTL3	F: AGATGGGTAGAAAGCCTCCT
METTL3	R: TGGTCAGCATAGGTTACAAGAGT
GAPDH	F: ACAACTTTGGTATCGTGGAAGG
GAPDH	R: GCCATCACGCCACAGTTTC

## Data Availability

Research data are not shared.

## Supplementary Materials

Supplementary Materials Supplementary Figure 1: the flow cytometric analysis of SCAPs to determine the MSC characteristics showed that CD90, CD105, and CD146 were positively expressed and CD45 and CD34 were negatively expressed in SCAPs. Supplementary Figure 2: miR-196b-5p mimic attenuated osteo/odontogenic differentiation promoted by METTL3 overexpression in SCAPs. (A) ALP activity assay demonstrating that the SCAP-Consh+METTL3 group was higher than that in the SCAP-Consh+Vector and METTL3+miR-196b-5p mimic groups. (B) Alizarin red staining analysis results demonstrating that the SCAP-Consh+METTL3 group was higher than that in the SCAP-Consh+Vector and METTL3+miR-196b-5p mimic groups. One-way ANOVA was used to analyze the statistical significance. All error bars signify standard deviations (*n* = 3). ^∗^*p* ≤ 0.05 and ^∗∗^*p* ≤ 0.01.
