# Supplementary material for: METTL3 Promotes Osteo/Odontogenic Differentiation of Stem Cells by Inhibiting miR-196b-5p Maturation
Source: Stem Cells Int. 2023 Jun 7;2023:8992284. doi: 10.1155/2023/8992284 (PMC10266913; doi:10.1155/2023/8992284)
Supplement: Supplementary Materials — Supplementary Figure 1: the flow cytometric analysis of SCAPs to determine the MSC characteristics showed that CD90, CD105, and CD146 were positively expressed and CD45 and CD34 were negatively expressed in SCAPs. Supplementary Figure 2: miR-196b-5p mimic attenuated osteo/odontogenic differentiation promoted by METTL3 overexpression in SCAPs. (A) ALP activity assay demonstrating that the SCAP-Consh+METTL3 group was higher than that in the SCAP-Consh+Vector and METTL3+miR-196b-5p mimic groups. (B) Alizarin red staining analysis results demonstrating that the SCAP-Consh+METTL3 group was higher than that in the SCAP-Consh+Vector and METTL3+miR-196b-5p mimic groups. One-way ANOVA was used to analyze the statistical significance. All error bars signify standard deviations (n = 3). ∗p ≤ 0.05 and ∗∗p ≤ 0.01. [file 8992284.f1.pdf]

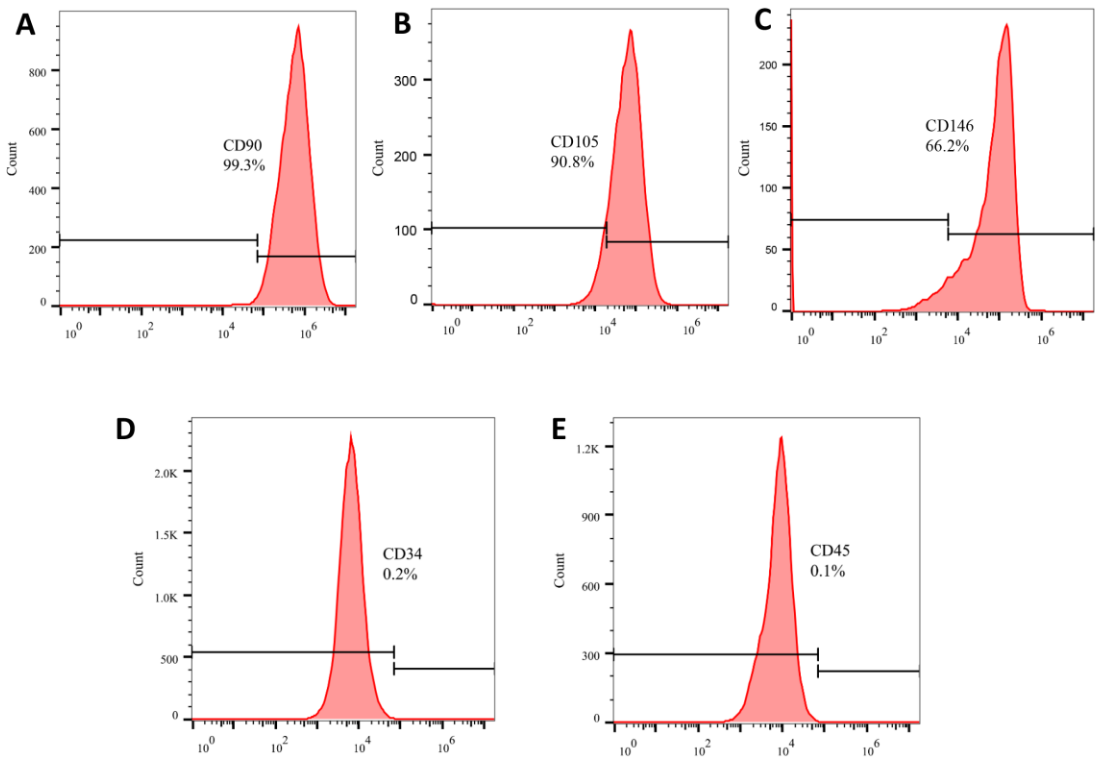


**Supplementary Fig. 1. The flow cytometric analysis of SCAPs to determine the MSCs characteristics showed that the CD90, CD105 and CD146 were positively and CD45 and CD34 were negatively expressed in SCAPs.**

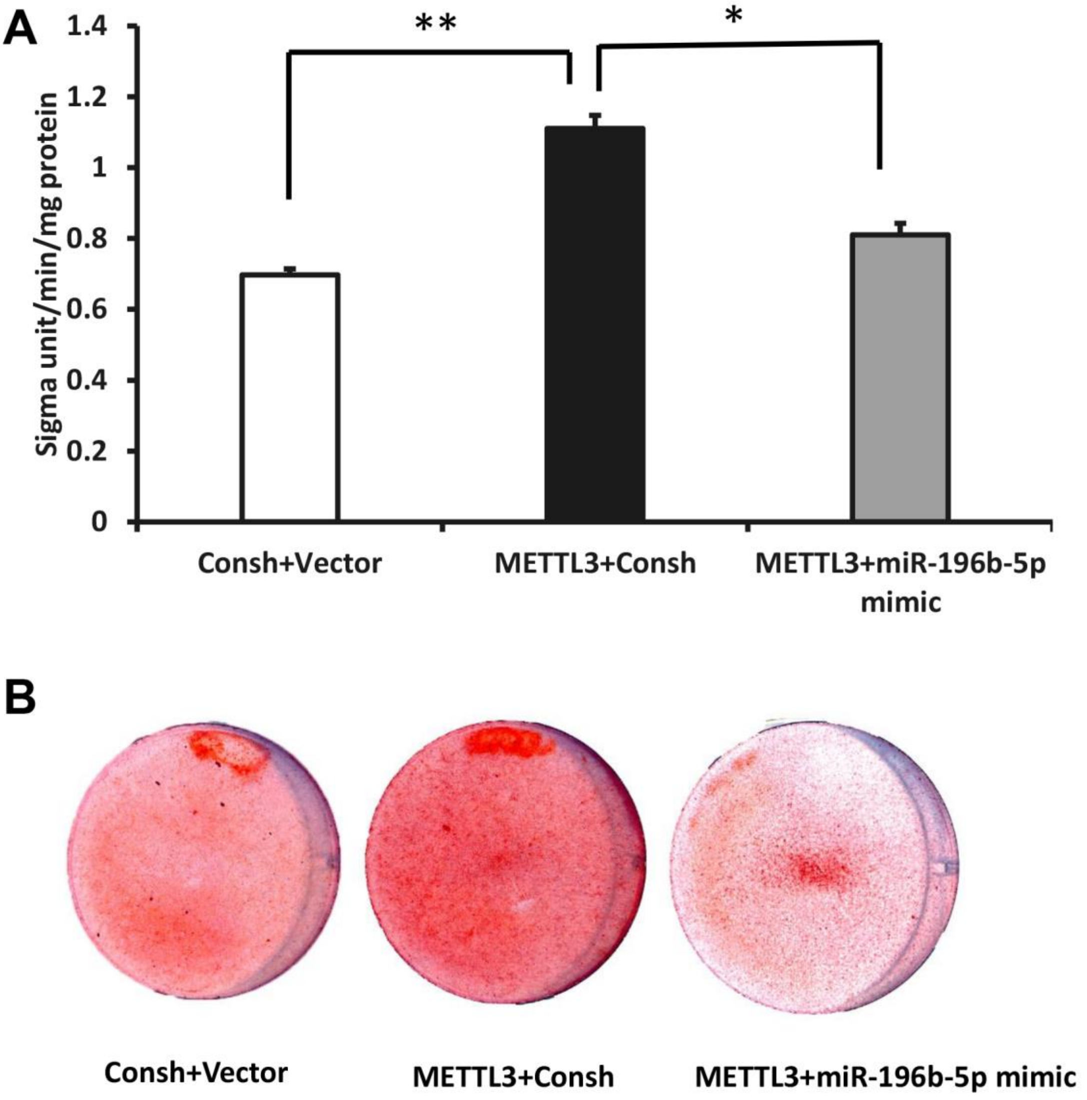


**Supplementary Fig. 2. miR-196b-5p mimic attenuated osteo-/dentinogenesis differentiation promoted by METTL3 overexpression in SCAPs. (A) ALP activity assay demonstrating that SCAPs-Consh+ METTL3 group was higher than that in the SCAPs - Consh + Vector and METTL3 + miR-196b-5p mimic group. (B) Alizarin red staining analysis results demonstrating that SCAPs-Consh+ METTL3 group was higher than that in the SCAPs - Consh + Vector and METTL3 + miR-196b-5p mimic group. One-way ANOVA was used to analyze the statistical significance. All error bars signify standard deviations (n=3). *p≤0.05, **p≤0.01.**
